# A Novel RGD-4C-Saporin Conjugate Inhibits Tumor Growth in Mouse Models of Bladder Cancer

**DOI:** 10.3389/fonc.2022.846958

**Published:** 2022-04-11

**Authors:** Stefania Zuppone, Chiara Assalini, Claudia Minici, Oronza A. Botrugno, Flavio Curnis, Massimo Degano, Angelo Corti, Francesco Montorsi, Andrea Salonia, Riccardo Vago

**Affiliations:** ^1^ Urological Research Institute, Division of Experimental Oncology, IRCCS San Raffaele Scientific Institute, Milano, Italy; ^2^ Biocrystallography Unit, Division of Immunology, Transplantation, and Infectious Diseases, IRCCS San Raffaele Scientific Institute, Milano, Italy; ^3^ Functional Genomics of Cancer Unit, Division of Experimental Oncology, IRCCS San Raffaele Scientific Institute, Milano, Italy; ^4^ Tumor Biology and Vascular Targeting Unit, Division of Experimental Oncology, IRCCS San Raffaele Scientific Institute, Milano, Italy; ^5^ Faculty of Medicine and Surgery, Università Vita-Salute San Raffaele, Milano, Italy

**Keywords:** saporin, recombinant protein, bladder cancer, RGD peptide, targeted therapy, ribosome inactivating proteins, RGD-integrins

## Abstract

Although toxin may have some advantages compared to chemotherapeutic drugs in cancer therapy, e.g. a potent cytotoxic activity and a reduced risk of resistance, their successful application in the treatments to solid tumors still remains to be fully demonstrated. In this study, we genetically modified the structure of the plant-derived single-chain ribosome inactivating protein saporin (SAP) by fusing its N-terminus to the ACDCRGDCFCG peptide (RGD-4C), an αv-integrin ligand, and explored the anti-tumor activity of the resulting protein (called RGD-SAP) *in vitro* and *in vivo*, using a model of muscle invasive bladder cancer. We found that the RGD-4C targeting domain enhances the cytotoxic activity of SAP against various tumor cell lines, in a manner dependent on αv-integrin expression levels. In a subcutaneous syngeneic model of bladder cancer, RGD-SAP significantly reduced tumor growth in a dose-dependent manner. Furthermore, systemic administration of RGD-SAP in combination with mitomycin C, a chemotherapeutic drug currently used to treat patients with bladder cancer, increased the survival of mice bearing orthotopic bladder cancer with no evidence of systemic toxicity. Overall, the results suggest that RGD-SAP represents an efficient drug that could be exploited, either alone or in combination with the state-of-the-art therapies, for the treatment of bladder cancer and, potentially, of other solid tumors.

## Introduction

Toxic molecules produced by plants have been assumed to be part of their defense weapons, even if in some cases a few of them are present in edible plants, including species that are eaten raw. From an evolutionary point of view, the selective pressure by the environment on plants has led to the development and optimization of highly efficient toxin molecules, which may represent potent and efficient cytotoxic agents that can be potentially exploited in cancer therapy. Their small size, high molecular stability, easy of production, high potency and direct cell-killing property make this class of cytotoxic agents very attractive for the development of new anti-cancer therapies (1–4). Among the various toxins so far studied for this purpose, the plant-derived type I ribosome inactivating proteins (RIPs) represent ideal candidates, owing to their high efficiency in irreversibly inhibiting protein translation and causing prompt cell death (2, 5). Furthermore, type I RIPs consist only of an N-glycosidase domain, lacking the lectin domain, typical of type II RIP, which bind galactose residues on the cell surface and facilitates the catalytic portion to enter the cell. This feature offers the opportunity to couple type I RIPs with a tumor-targeting ligand that enable specific and selective delivery of the toxins to cancer cells, thereby improving their therapeutic index. According to this view, type I RIPs have been coupled to growth factors or other polypeptides capable of recognizing receptors over-expressed on the surface of cancer cells or on tumor endothelial cells (2, 6–8).

Saporin (SAP), a type I RIP characterized by unusual resistance to high temperature, denaturation and proteolysis and by a strong intrinsic cytotoxic activity, may represent a suitable candidate for the design and development of new anti-cancer drugs. Recent studies have shown that coupling SAP with tumor targeting ligands, such as monoclonal antibodies, peptides and aptamers, improves its cytotoxic effects on different cancer types, both *vitro* and *in vivo* (9–14). In particular, SAP-based, chimeric recombinant proteins formed by the toxin fused to the amino-terminal fragment of urokinase (11, 13), the epidermal growth factor (12, 15), the anti-CD22 ScFv (9) have been produced and successfully tested. Thus, the development of new strategies for targeted delivery of SAP to tumors is of great experimental and pharmacological interest. At this regard, a growing body of evidence suggests that integrins may represent important molecular targets on cancer cells. In fact, the expression of certain integrins is increased on various types of cancer cells and tumor vasculature, to regulate many steps of tumor progression, such as angiogenesis and tumor cell growth, survival, migration and invasion (16–19). For example, certain αv-integrins, such as αvβ3, αvβ6, α5β1 and αvβ5, are upregulated in various solid cancers, tumor microenvironment and upon anti-cancer therapy, while they are expressed at lower or undetectable levels in normal tissues (20). In particular, αvβ3 and αvβ5 are known to be overexpressed in the tumor vasculature and to be involved in tumor angiogenesis (21, 22). Integrin over-expression is associated with pathological outcomes including disease stage, metastasis formation, treatment resistance, and patient survival (20). Thus, ligands of specific integrin subclasses may be exploited, in principle, for the development of new tumor-homing derivatives of SAP.

In the last years, many investigators have explored the potential of peptides as integrin ligands, a promising class of molecules that, owing to their small size, low immunogenicity, ease of manufacture at reasonable costs, can overcome many of the limitations related to the use of monoclonal antibodies as targeting moieties. For instance, RGD-based peptides have been widely investigated as ligands for targeted delivery of drugs and nanoparticles to tumors. In particular, ACDCRGDCFCG (RGD-4C), a peptide capable of recognizing with high affinity αvβ3, and, although with a lower affinity, also αvβ5, α5β1 and αvβ6 (26) has proven useful to enhance the selective delivery of various types of compounds to tumors, including cytokines and toxins (23–26).

Based on these notions, we tested the hypothesis that fusing RGD-4C to SAP, by recombinant DNA technology, can increase its tumor selectivity and therapeutic activity. We show that the RGD-SAP conjugate can be easily produced in *E.coli* with no need of renaturation, and that this product can kill integrin-expressing cells more efficiently than a SAP variant lacking the RGD domain. Moreover, we show that RGD-SAP can inhibit the tumor growth in mouse models of bladder cancer.

## Materials and Methods

### Cell Cultures

Human bladder RT4, RT112, 5637 were maintained in RPMI 1640 supplemented with 10% fetal calf serum (FCS), 2 mM L-glutamine and antibiotics (100 U/mL penicillin and 100 µg/mL streptomycine-sulphate); breast MDA-MB 468 and glioblastoma U87 cancer cell lines as well as skin fibroblast cells were maintained in DMEM supplemented with 10% FCS, 2 mM L-glutamine and antibiotics (100 U/mL penicillin and 100 μg/mL streptomycine-sulphate). Murine MB49 bladder cancer cells were cultured in DMEM, supplemented with 10% FCS, 2 mM L-glutamine, antibiotics (100 U/mL penicillin and 100 µg/mL streptomycine-sulphate) and 1 mM sodium pyruvate.

MB49 Luc cells stably expressing luciferase were generated by transduction with a 3^rd^ generation lentiviral vector carrying the luciferase gene. pLenti PGK V5-LUC Neo (w623-2) was a gift from Eric Campeau, University of Massachusetts Medical School, Worcester, Massachusetts, US (Addgene plasmid # 21471). For lentivirus production, a monolayer of HEK293T cells, cultured in 10 cm^2^ dish, were incubated with the following mixture: transfer vector (10 μg), packaging vector Δr 8.74 (6.5 μg), Env VSV-G vector (3.5 µg), REV vector (2.5 μg) in 450 μl double distilled water, 50 μl calcium chloride (2.5 M) and 500 μl Hank’s buffered saline (2-fold). Sixteen hours later, the medium was replaced with culture medium and 24 hours later the medium was collected and 0.22 µm-filtered to recover virus particles. Virus particles were then used to transduce MB49 cells. Infected cells were then cultured in presence of G418 antibiotic (0.5 mg/ml) for fifteen days.

### Cloning of RGD-SAP and CYS-SAP in pET22b Vector

SAP fused with ACDCRGDCFCG or CGGSGG at its N-terminus were prepared by GenScript (New Jersey, USA). The nucleotide sequences were obtained from the corresponding amino acid sequences of saporin S and optimized for the expression in *E.coli*. A GGSSRSS sequence was interposed between ACDCRGDCFC and SAP as a spacer and a 6xHis tag was added at the C-terminus to allow the purification by affinity chromatography. The whole encoding sequences were inserted into the pET22b(+) vector (Novagen), forming the pET22b(+)-RGD-SAP (5′-NdeI- ACDCRGDCFCG-GGSSRSS-SAP-HHHHHH-EcoRI-3′) and the pET22b(+)-CYS-SAP (5′-NdeI-CGGSGG-SAP-HHHHHH-EcoRI-3′) expression vectors. Ligation products were used to transformed the *E.coli* strain DH5alpha (Invitrogen).

### Expression and Purification of RGD-SAP and CYS-SAP

The expression of RGD-SAP and CYS-SAP in transformed BL21(DE3) *E.coli* cells (Novagen) was induced for 3 hours at 37°C with 0.1 mM IPTG. Bacterial pellet from 1 L culture was resuspended in 15 ml of 50 mM Tris-HCl, pH 7.5, supplemented with a cocktail of protease inhibitor (Sigma-Aldrich). Soon after, 10 mM of imidazole, lysozyme (250 µg/ml) and DNAse (20 µg/ml) were added. The bacterial solution was then incubated on ice for 45 min, subjected to 3 cycles of sonication (using a UW3100 Bandelin sonicator operating at 60% power; 2 min cycle duration with 1 sec pulse and 1 sec pause), and centrifuged at 4°C for 25 min at 10000 x g. Soluble RGD-SAP and CYS-SAP contained in the supernatant were then purified by metal chelate affinity chromatography using a HisTrap HP 5 ml column (GE Healthcare Life Sciences) equilibrated in Tris-HCl 50 mM pH 7.5, 300 mM NaCl supplemented with 10 mM imidazole (and 5 mM DTT for CYS-SAP) operated with the AktaPurifier10 FPLC system. To elute proteins, imidazole concentration was increased step by step up to 500 mM in 20 column volumes (CV). The fractions containing the target proteins were dialyzed against 50 mM Tris-HCl, pH 7.5 (CYS-SAP) or 50 mM bicine, pH 8.2 (RGD-SAP) at 4°C for 16 hours. A cation exchange chromatography on HiTrap SP Sepharose FF column (GE Healthcare Life Sciences) was then performed for further purification, using a 20 CV gradient up to 1 M NaCl for protein elution. The proteins were then concentrated using 10 KDa cutoff Amicon centrifugal filters (Millipore-Sigma) and dialyzed against PBS with slide A lyzer dialysis cassette (Thermo Fisher). All solutions used in purification steps were prepared with sterile and endotoxin-free water (S.A.L.F. Laboratorio Farmacologico SpA, Bergamo, Italy). Protein concentration was measured using the BCA Protein Assay DC^™^ Kit (BioRad). Protein purity and identity was checked by SDS-PAGE and Western blotting. Both CYS-SAP and RGD-SAP showed comparable yields ranging from 0.6 to 1.2 mg/l of bacterial culture.

### Western Blot Analysis

Cells were washed twice with cold PBS, collected by scraping and centrifuged 5 min at 300 g. Cells were lysed for 30 min on ice in ice-cold buffer (50 mM Tris-HCl, pH 7.5, containing 150 mM NaCl, 2 mM NaF, 1 mM EDTA, 1 mM EGTA, 1 mM Na_3_VO_4_,1 mM PMSF, 75 mU/ml aprotinin (Sigma-Aldrich), 1% TritonX-100 and a proteinase inhibitor cocktail^®^ (Sigma-Aldrich). Cell lysates were centrifuged at 10000 x g at 4°C for 10 min and the supernatants were recovered and quantified for total protein content. Equal amounts of cell protein extracts were separated by SDS-PAGE under reducing conditions unless stated otherwise. For western blot analysis, proteins were transferred onto a nitrocellulose membrane, incubated with 5% non-fat powdered milk in TBS-T (0.5% Tween-20) for 1 hour and then with the following antibodies: anti-saporin anti-serum (rabbit, 1:5000), anti-caspase 3 (rabbit, 1:2000, clone E87, Abcam), anti-beta actin (mouse, 1:10000, clone AC-15, Sigma-Aldrich). The antibody binding was detected using a secondary horseradish peroxidase conjugated antibodies (donkey anti-mouse/rabbit IgG HRP-linked, GE Healthcare) and an enhanced chemiluminescent (ECL, Merck Millipore).

Seed SAP used as positive control for Western blot analysis was purchased from Advanced Targeting Systems, which had purified it from the seeds of the Soapwort plant (*Saponaria officinalis*).

### Flow Cytometry Analysis

Cultured cell lines were detached by TripLE Express (Gibco) to preserve receptor integrity, washed with PBS containing 1% FCS and incubated with PE-conjugated Ab specific for human αvβ3 and αvβ5 (R&D System) and FITC-conjugated Ab specific for human αvβ6 integrins (NovusBio). For receptor detection, cells were incubated with the fluorescently labelled Ab at 4°C for 30 min. Stained cells were resuspended in 100 μL of PBS containing 1% FCS. Samples were run through an Accuri™ flow cytometer (BD Biosciences). All data were analysed by FCS Express and expressed as relative fluorescence intensity (RFI), calculated as follows: mean fluorescence intensity after mAb staining/mean fluorescence intensity after isotype-negative control staining. Analysis was done on 20,000 gated events acquired per sample.

### Cell Viability Assay (MTT)

Cultured cell lines (5 x 10^3^ cells/well) were seeded in 96 wells plates and incubated for 72 h with various amounts of RGD-SAP or CYS-SAP at 37°C, 5% CO_2_. Cell viability was then quantified by 3-(4,5-dimethylthiazol-2-yl)-2,5-diphenyltetrazolium bromide staining (MTT) (working solution 0.5 µg/ml). After 1 h of incubation, the supernatants were removed, the formazan crystals were dissolved with dimethyl sulfoxide and the absorbance at 570 nm was measured using a microtiter plate reader. Competitive experiments were performed as described above using 100 nM of RGD-SAP or 1000 nM of CYS-SAP in the presence of 5000 nM of ACDCRGDCFCG peptide for 48 and 72 hours.

To induce caspase 3 activation, cells were treated with 30 nM RGD-SAP or 2 mM DTT for 48 and 72 hours.

### *In Vivo* Studies

Studies on animal models were approved by the Institutional Animal Care and Use Committee (Institutional Animal Care and Use Committee, IACUC) and performed according to the prescribed guidelines. C57BL/6 female mice (7 weeks old, Charles River, Calco, Italy) were challenged with subcutaneous injection in the left flank of 3-5 x 105 MB49 living cells; 5 days later, 4-6 mice/group were intravenously administered with various doses of RGD-SAP or CYS-SAP diluted in sodium chloride (0.9%, i.v., 200 µl). Tumor growth was estimated by calculating the volume using the formula r1 × r2 × r3 × 4/3 π, where r1 and r2 are the longitudinal and lateral radii, and r3 is the thickness of tumors protruding from the surface of normal skin. Animals were euthanized when tumors reached 10 mm in diameter, became ulcerated or a 15% animal body weight loss was measured.

Orthotopic syngeneic tumor were developed according to the procedure described by Loskog et al. (27). Briefly, C57BL/6 female mice were anesthetized with ketamine/xylazine (80/10 mg/kg) and catheterized (PE50 catheter, BD Biosciences) using lubricated catheters with 2.5% lidocaine-containing gel (Luan). To enhance tumor engraftment, 100 µl of poly-L-lysine (0.1 mg/ml, mw 70000–150000, Sigma Aldrich), was injected transurethrally into the bladder and left in place for 30 min, then bladder was washed with PBS and instilled with 5x104 MB49 Luc diluted in serum-free medium (100 µl/mice), 30 min later the catheters were removed. On day 7, mice were treated with mitomycin C (MMC) alone (n=10), administered transurethrally and kept in the bladder for 1 hour (50 µg in 100 µl of PBS, every 4 days for 2 times), or combined with RGD-SAP (n=5) or RGD-SAP alone (i.v., 200 µl, every 5 days, for 3 times) (n=10), starting at day 9. Control group of mice was treated with vehicle (sodium chloride, 0.9%, i.v., 200 µl) (n=10). Orthotopic tumor engraftment and growth was monitored once a week by *in vivo* bioluminescence imaging (IVIS). Tumor growth was estimated by acquiring the bioluminescence signal (BLI) and it expressed in total photon flux. Mice were euthanized when the BLI intensity suddenly drop, due to irreversible necrosis and accompanied with hematuria or animal lethargy.

### Blood Sample Collection and Biochemical Parameters Analysis

Blood samples were collected from the retroorbital plexus of anesthetized mice using 4% isoflurane at the end of each experiment or before animal sacrifice. Blood samples were left at room temperature for at least 30 min before being processed and then centrifuged (800 x g, 10 min) for serum separation. Serum albumin, aspartate transaminase, alanine transaminase, creatinine and urea were determined by using an automated analyzer (ScilVet ABC plus and Idexx Procyte analyzers) according to the manufacturers’ instructions. Standard controls were run before each determination.

### Statistical Analysis

All *in vitro* experiments were performed at least in triplicate. Mouse experiments were performed using at least 4 mice per group. When appropriate, statistical significance was determined using a 2-tailed Student’s *t* test. For tumor growth analyses, we performed one-way ANOVA statistical analysis. Survival curves were compared using the log rank test. Tests symbols mean: *p < 0.05; **p < 0.01; ***p < 0.001; ns, not significant.

## Results

### Production and Characterization of RGD-SAP and CYS-SAP

**Figure 1:**
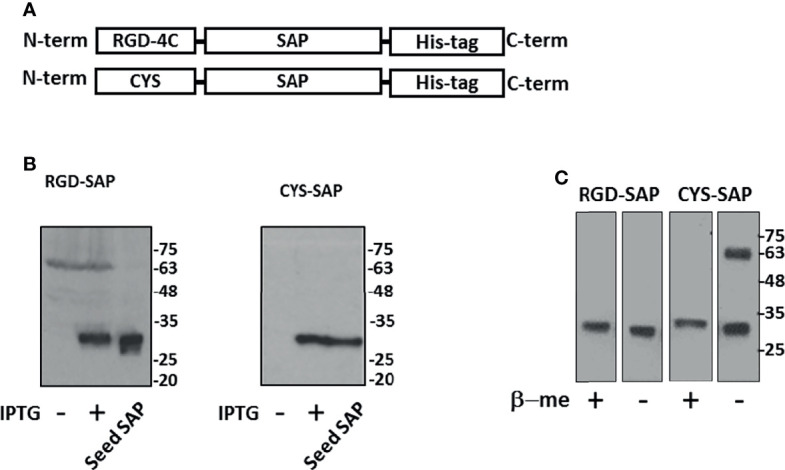
Design, expression and purification of RGD-SAP and CYS-SAP recombinant proteins. **(A)** Schematic representation of RGD-SAP and CYS-SAP recombinant proteins. The RGD (ACDCRGDCFCG) targeting and CYS (CGGSGG) non-targeting peptides were inserted by genetic recombination at the N-terminus of SAP. A histidine (His)-tag was added at the C-terminus of the two constructs to aid in the purification of the recombinant proteins. **(B)** Effect of IPTG on RGD-SAP and CYS-SAP expression in BL21DE3 *E.coli* cells as determined by western blot using an anti-SAP antibody. IPTG (+) and (-), induced and not induced cells, respectively. Seed-derived SAP (200 ng) was used as positive control for WB analysis. **(C)** WB analysis of purified RGD-SAP and CYS-SAP under reducing (β-mercaproethanol (β-me) +) or non-reducing (β-me -) conditions.

RGD-SAP, consisting of RGD-4C fused to the N-terminus of SAP was produced in *E.coli* cells by recombinant DNA technology. In parallel we have also produced a SAP variant with a Cys residue in place of the RGD-4C domain (CYS-SAP) (**Figures 1A, B**).

To facilitate their purification and to promote endosomal escape of the toxin into recipient cells, both products were genetically engineered to express a histidine tag at the C-terminus (28). Since SAP can inactivate prokaryotic ribosomes, their production in *E.coli* was induced with IPTG for only 3 h. Western blot analysis of the purified RGD-SAP, performed under reducing and non-reducing conditions, showed a single band of 30 kDa as expected for monomers (**Figure 1C**), suggesting that inter-chain disulfide bonds between the ACDCRGDCFCG domains of different molecules were not formed. In contrast, two bands corresponding to monomers and dimers were detected in CYS-SAP, suggesting that an inter-chain disulfide bond was formed between the N-terminal cysteines of this protein (**Figure 1C**).

### RGD-SAP Can Kill Integrin-Expressing Cells More Efficiently Than CYS-SAP

**Figure 2:**
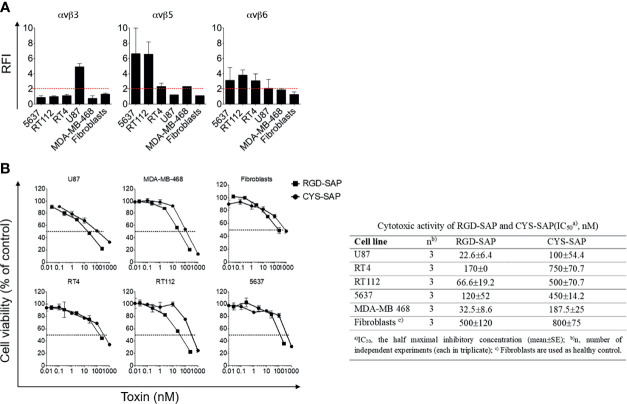
Integrins expression on cancer cell lines and *in vitro* biological activity evaluation of RGD-SAP and CYS-SAP recombinant proteins. **(A)** 5637, RT112, RT4, U87, MDA-MB-468 cancer cell lines and fibroblasts were analyzed by flow cytometry for αvβ3, αvβ5 and αvβ6 integrins expression. Relative Fluorescence Intensity (RFI) are shown as mean±SD. The dashed red line represents the threshold arbitrarily defining positive expression (RFI=2). **(B)** Effect of various amounts of RGD-SAP or CYS-SAP on the indicated cells as determined by MTT assay after 72 h treatment. One representative experiment out of three performed is shown. Cell viability is expressed as percentage to untreated cells (mean ± SD). The IC_50_ from three different experiments is reported as mean ± SE.

RGD-4C can recognize αv-integrins with different affinities (29). Integrins, such as αvβ3, αvβ5 and αvβ6, are present on a variety of tumor cells (24–26, 30). Therefore, to identify tumor cells that could be exploited as targets to validate the targeting properties of RGD-SAP *in vitro*, we characterized the surface expression of integrins by various cancer cell lines, including U87 glioblastoma cells, RT4, RT112 and 5637 bladder cancer cells, MDA-MB-468 breast cancer cells and normal fibroblasts, by flow cytometry. The results showed that U87 cells express high levels of αvβ3, but not of αvβ5 and αvβ6, whereas RT4, RT112 and 5637 and MDA-MB-468 cells showed a moderate-high positivity for αvβ5 and αvβ6, but not of αvβ3. Normal fibroblasts expressed none of these integrins (**Figure 2A**).

To assess whether the RGD domain can increase the cytotoxic effects of saporin against cancer cells, we then tested the cytotoxic effects of RGD-SAP and CYS-SAP on these cell lines. A stronger cytotoxic effect of RGD-SAP, compared to CYS-SAP, was observed with all cancer cells, but not with normal fibroblasts (**Figure 2B**). Considering that fibroblasts do not express αvβ3 and αvβ5, i.e. integrins known to be recognized by RGD-4C, these data suggest that the stronger cytotoxic effects of RGD-SAP against cancer cells was mediated by an RGD-dependent targeting mechanism.

**Figure 3:**
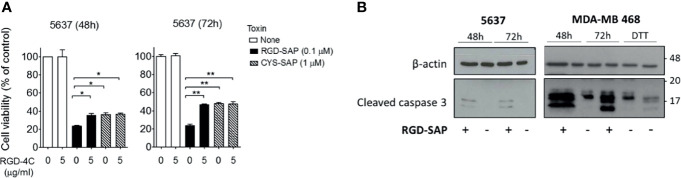
RGD-SAP target specificity and caspase 3 activation. **(A)** 5637 bladder cancer cells were incubated with RGD-SAP (0.1 µM) or CYS-SAP (1 µM) in the presence or absence of ACDCRGDCFC peptide (5 µM). The cell viability was evaluated after 48 and 72 h by MTT assay. Results are shown as mean ± SE. *p < 0.05, **p < 0.01 by non-parametric unpaired *t* test. **(B)** The activation of apoptosis *via* caspase 3 cleavage upon RGD-SAP (30 nM) treatment was evaluated in 5637 and MDA-MB-468 cells by analyzing the intracellular levels of caspase-3. Cells were incubated with RGD-SAP for 48 and 72 h and cell lysates were analyzed by western blot with anti-caspase-3 or an anti-β-actin antibodies for protein normalization. DTT (2 mM) was included as positive control for caspase-3 activation.

To verify this hypothesis, we performed competition experiments with the free RGD-4C peptide on 5637 cell line, selectively sensitive to RGD-SAP and representative of a human muscle-invasive model of bladder cancer. To this end, cells were treated with 0.1 µM RGD-SAP or 1 µM CYS-SAP, concentrations reflecting the different sensitivity of cells towards the two toxins, in the presence or absence of an excess of free RGD-4C. As expected, RGD-4C significantly decreased the activity of RGD-SAP, but not that of CYS-SAP (**Figure 3A**). This result lends support to the hypothesis that indeed the RGD domain of RGD-SAP contribute to the cytotoxic activity of this conjugate by a receptor-mediated targeting mechanism, likely involving integrins.

It is well known that SAP induces cell apoptosis. Thus, we then investigated the activation of programmed cell death by analyzing caspase 3 in cells treated with RGD-SAP. To this aim, 5637 and MDA-MB-468 epithelial cells were incubated with RGD-SAP or DTT, a positive control, for 48 and 72 h. As shown in **Figure 3B**, caspase 3 activation was detectable as cleaved form in all samples treated with the toxin. These and the above results indicate that fusing the RGD moiety to SAP enhances the delivery and uptake of the toxin.

### RGD-SAP Is Endowed With Antitumor Activity on a Subcutaneous Model of Bladder Cancer

**Figure 4:**
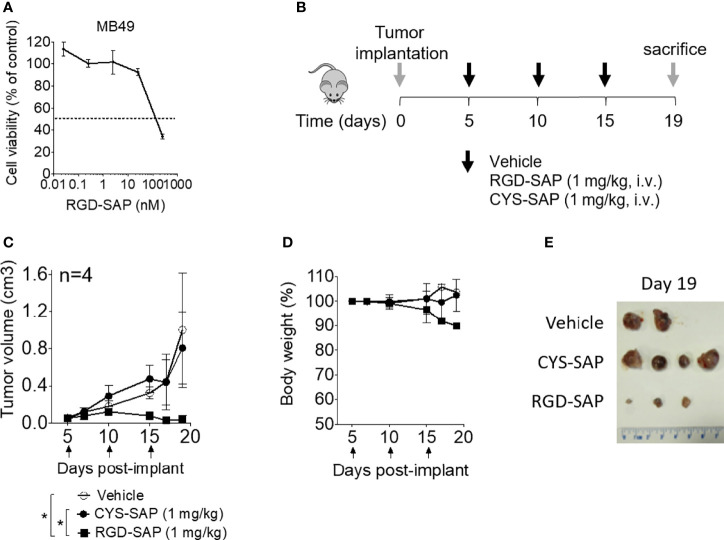
Effects of RGD-SAP and CYS-SAP on tumor growth in a subcutaneous syngeneic bladder cancer mouse model. **(A)** Effect of RGD-SAP on MB49 murine bladder cancer cell line viability as measured by MTT assay. **(B)** Schematic representation of the experimental design and treatment schedule. MB49 cells were subcutaneously implanted in 7-week-old C57BL/6 WT female mice. Tumor bearing-mice were treated i.v. with the indicated doses of CYS-SAP or RGD-SAP or vehicle every 5 days after tumor implantation (arrows). **(C)** Effect of 1 mg/kg dose of CYS-SAP or RGD-SAP on MB49 bladder cancer growth. Tumor volumes (mean ± SD, n=4 mice/group) are shown. *p < 0.05, by one-way ANOVA test. **(D)** Effect of treatments on the animal body weight change. Animal weights are reported as percent of starting body weight (mean ± SD of 4 animals per group). **(E)** A representative image of MB49 subcutaneous tumors explanted from mice that survived to the end of the experiment (day 19): (vehicle (n=2), 2 mice were sacrificed at day 16 due to tumor ulceration; RGD-SAP (n=4), 1 tumor undetectable).

The anti-tumor activity of RGD-SAP and CYS-SAP were then investigated using C57BL/6 mice bearing subcutaneous murine MB49 urothelial carcinoma cells. A preliminary experiment, performed *in vitro*, showed that RGD-SAP could kill these cells (**Figure 4A**).

Thus, mice were treated, systemically, with 1 mg/kg of RGD-SAP or CYS-SAP at day 5. The treatment was repeated three times every five days (**Figure 4B**). RGD-SAP could reduce tumor growth after the second administration, leading to marked reduction of the tumor volume in all mice and complete eradication in one mouse (**Figures 4C–E** and **Supplementary Figure 1A**). On the contrary, CYS-SAP did not show any effect on tumor growth, suggesting that the therapeutic effect of RGD-SAP crucially involved the RGD domain. However, the dose of RGD-SAP used in this experiment also caused loss of body weight at day 19 (**Figure 4D**) and severe necrosis in the tails where the drug was injected.

**Figure 5:**
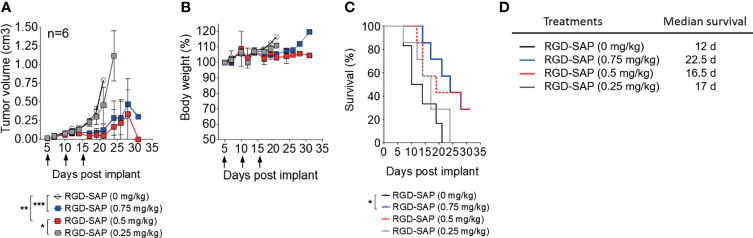
Dose dependent effects of RGD-SAP on tumor growth in a subcutaneous syngeneic bladder cancer mouse model. **(A)** MB49 cells were subcutaneously implanted in 7-week-old C57BL/6 WT female mice (n=6/group) were treated i.v. with the indicated doses of RGD-SAP every 5 days after tumor implantation for three times (arrows). Tumor volumes (mean ± SD) are shown. *p < 0.05; **p < 0.01; ***p < 0.001 by using one-way ANOVA test. **(B)** Animal weights are reported as percent of starting body weight (mean ± SD of 6 animals per group). **(C)** Kaplan–Meyer plot of animal survival and **(D)** the associated median survival time. Results from a Mantel–Cox two-sided log-rank test are shown when statistically significant (*p < 0.05) for RGD-SAP 0.75 mg/kg (blue line, hazard ratio 5.25; 95% CI, 1.19–23.10) versus vehicle (black line).

To determine the minimal effective, non-toxic dose of RGD-SAP tumor-bearing mice were treated with 0.75, 0.5, 0.25 mg/kg of RGD-SAP at days 5, 10, 15 after tumor implantation, as described above. The doses of 0.75 and 0.5, but not 0.25 mg/kg, caused a significant delay of tumor growth, pointing to a dose-dependent effect (**Figure 5A** and **Supplementary Figure 1B**), without causing loss of body weight (**Figures 5B–D**).

**Figure 6:**
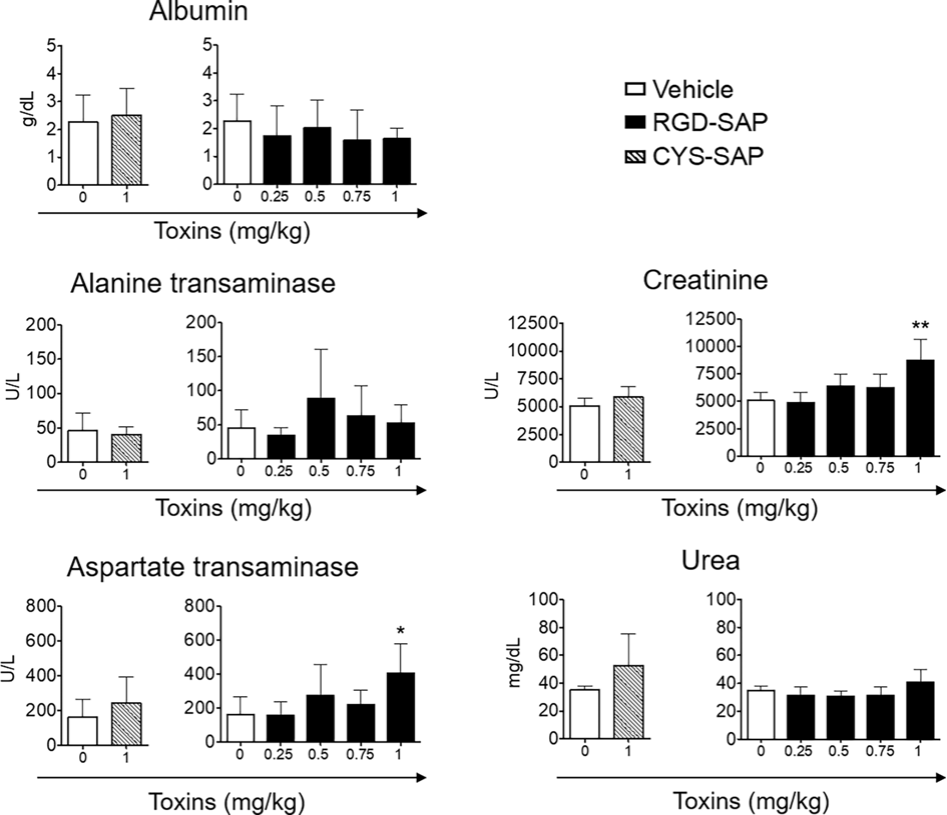
Comparison of clinical biochemistry parameters in mice treated with CYS-SAP or different RGD-SAP concentrations. Blood samples were collected from treated mice and the concentration of albumin, alanine transaminase, aspartate transaminase, creatinine and urea in the serum were measured. Data are shown as mean ± SD from n = 4/6 mice per condition. Results from unpaired Student *t* test are shown. All treatments were compared to vehicle (*p < 0.05; **p < 0.01).

The toxicological effects RGD-SAP was further investigated. To this aim, we collected blood samples at the end of each experiment or before animal sacrifice and analyzed biochemical parameters of liver and kidney toxicity (albumin, alanine transaminase, aspartate transaminase for liver toxicity, and creatinine and urea for kidney toxicity). As shown in **Figure 6**, RGD-SAP systemic toxicity was dose dependent and apparent only with the highest dose used (1 mg/kg). In fact, significantly higher values of AST and CHE were measured in mice treated with 1 mg/kg RGD-SAP, but not with the lower doses.

### RGD-SAP Can Delay Tumor Growth in an Orthotopic Mouse Model of Syngeneic Bladder Cancer

**Figure 7:**
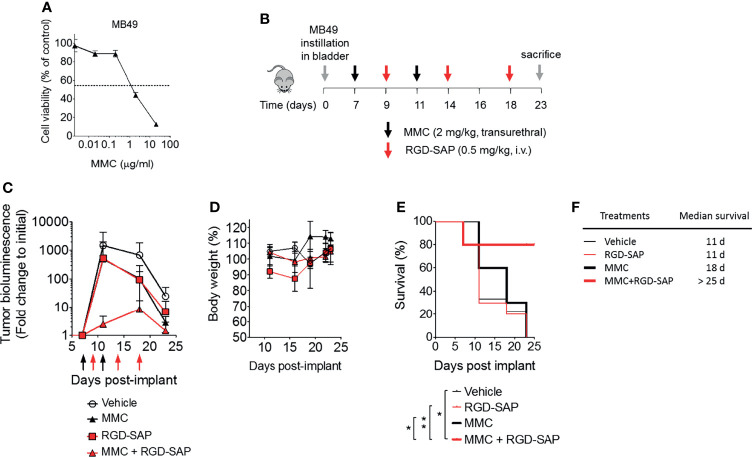
Effects of MMC and RGD-SAP on tumor growth in an orthotopic syngeneic bladder cancer mouse model. **(A)** Effect of mitomycin C (MMC) on MB49 murine bladder cancer cell line viability as measured by MTT assay. **(B)** Schematic representation of the *in vivo* experimental design and treatment schedule. Luciferase-expressing MB49 cells were transurethrally injected into the bladder of 7-week-old C57BL/6 female mice. Tumor engraftment was monitored by *in vivo* bioluminescence imaging. Tumor bearing mice were randomized into 4 experimental groups and treated with the indicated dose of MMC (transurethral, black arrows, n=10 mice), RGD-SAP (i.v., red arrows, n=10 mice), MMC + RGD-SAP (n=5 mice) or vehicle (n=10 mice). The results of two experiments with 5 animals/group were considered and cumulated **(C)** Tumor growth (mean ± SD) as determined over time by fold change to the initial detectable bioluminescence. **(D)** Effect of treatments on the animal body weight change. Animal weights are reported as percent of starting body weight (mean ± SD of 5-10 animals per group). **(E)** Kaplan–Meyer plot of animal survival and **(F) **median survival time. Results from a Mantel–Cox two-sided log-rank test are shown when statistically significant (*p < 0.05, **p < 0.01) for MMC+RGD-SAP (bold red line) versus vehicle (thick black line, hazard ratio 7.19; 95% CI, 1.51–34.26) or MMC (bold black line, hazard ratio 5.96, 95% CI, 1.44-24.69) or RGD-SAP (bold red line, hazard ratio 0.15, 95% CI, 0.03-0.62).

The anti-tumor efficacy of RGD-SAP was then investigated in an orthotopic model of urothelial carcinoma. To this aim, we genetically engineered MB49 cells to express luciferase (MB49-luc). MB49-luc cells were then orthotopically implanted into immunocompetent C57BL/6 mice. Tumor engraftment and growth, as monitored by *in vivo* bioluminescence imaging, occurred in 100% of mice in 5-7 days after cells inoculation. This tumor model resembles advanced bladder cancer and is characterized by high proliferation rate accompanied by protrusion of the mass into the bladder lumen, obstruction of urethra, hematuria and necrosis in the tumor core a few days upon engraftment (27). These features can lead to an inadequate drug delivery to the tumor mass (27). To overcome this limitation, we decided to use mitomycin C (MMC) as a tool to slow down the tumor growth and delay necrosis formation, thereby allowing the toxin to reach tumor cells and exert its specific effect. MMC is one of the most widely used agents for preventing recurrence of superficial bladder cancer in clinics, usually administered intravesically after transurethral resection of cancer lesions (31–33). Of note, MB49 bladder cancer cells were extremely sensitive to MMC (IC50 ~2 μg/ml) (**Figure 7A**).

**Figure 8:**
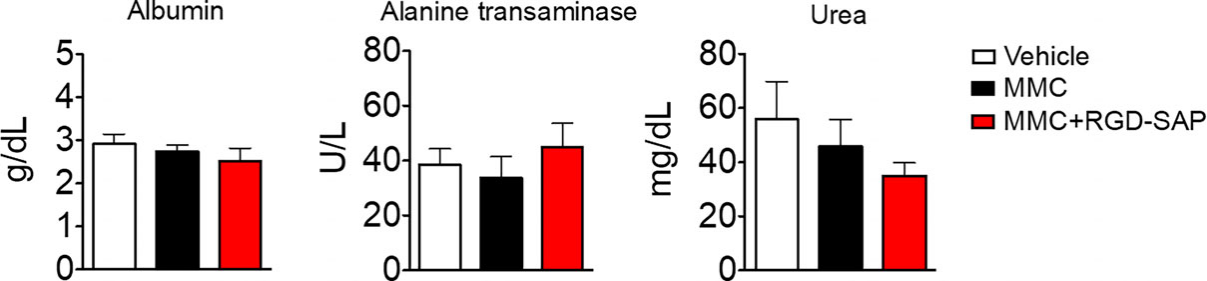
Comparison of clinical biochemistry parameters in mice treated with mitomycin C (MMC) alone or in combination with RGD-SAP. Blood samples were collected from treated mice and the concentration of albumin, alanine transaminase and urea in the serum were measured. Values are shown as mean ± SD from n = 10 for vehicle and MMC; n=5 for MMC + RGD-SAP.

At the time of tumor detection (day 7 after cells infusion into the bladder) two experimental groups were treated with MMC through transurethral administration. A second dose of MMC was given after four days. In between, mice received a first dose of RGD-SAP (systemically, 0.5 mg/kg) or vehicle, which was repeated for three times (**Figure 7B**). One group of mice was treated with RGD-SAP alone, as control. As previously demonstrated (34), in this model, after an initial increase, the bioluminescent signal drops in a time dependent manner, due to the reduced light emission from extended necrotic and hemorrhagic areas compared with vital tumor areas. In the control group the bioluminescent signal increased, reaching the maximum value after 11 days, and then started decreasing. Although a similar behavior was observed after treatment with MMC or RGD-SAP alone, the combination of these drugs greatly limited the increase of the signal (**Figure 7C**), which implies a relevant anti-tumor effect. Accordingly, a significant effect on mice survival was observed in the group of mice treated with RGD-SAP/MMC combination (**Figures 7E, F**), as 80% of mice were still alive when the experiment ended after 23 days. Histochemical analysis of the tumors, explanted after mouse scarification, showed that only one mouse treated with the combined therapy had a necrotic tumor, while all the others did not display any necrotic area, suggesting that this treatment consistently reduced the tumor growth. Furthermore, no evidence of toxicity was observed, judging from the lack of significant changes of body weight (**Figure 7D**), as well as of albumin, alanine transaminase or urea levels in the bloodstream (**Figure 8**).

## Discussion

The results show that the fusion of RGD-4C with SAP enables selective delivery of this toxin to tumors, thereby enhancing its antitumor activity. In particular, the results show that RGD-SAP can kill cells expressing the integrins αvβ3, αvβ5 and αvβ6 more efficiently than CYS-SAP, a control conjugate lacking the RGD-4C domain. As expected, the improved cytotoxic activity of RGD-SAP was inhibited, *in vitro*, by an excess of free RGD-4C peptide. Considering the known ability of RGD-4C to bind αvβ3 (affinity value: 8.3 nM), αvβ5 (46 nM), α5β1 (244 nM) and αvβ6 (380 nM) integrins (26), although with different affinities, and the known overexpression of these integrins in tumors, these findings suggest that integrin targeting was an important mechanism that contribute to the improved activity of RGD-SAP. We tested the expression of αvβ3, αvβ5 and αvβ6, to associate the RGD-SAP cytotoxicity to the integrin expression on target tumor cells. U87, which expresses the highest amount of αvβ3, are the most sensitive to RGD-SAP, but also other cell lines, expressing αvβ5 and αvβ6 are enough sensitive to RGD-SAP, considerably more than the untargeted CYS-SAP. It is likely that not only αvβ3, but also the other abovementioned integrins can contribute to the RGD-SAP cytotoxicity. In addition, we cannot exclude contribution of other RGD-interacting integrins to RGD-SAP cytotoxicity. To lower the risk that RGD-4C fusion with saporin could reduce or abolish the binding of the peptide to integrins, e.g. by steric hindrance, we have introduced a seven amino acids flexible linker. The higher activity of RGD-SAP with respect to untargeted SAP and the reduction of its cytotoxicity upon competition with RGD-4C peptide, suggests that with this linker RGD-4C preserved, at least partially if not at all, its functional properties after coupling to saporin. The results of *in vivo* studies in different mouse models of bladder cancer show that RGD-SAP can reduce tumor growth and significantly prolong animal survival without inducing detectable side effects. These results and the notion that RGD-4C is a compound with a proven utility as ligand for the targeted delivery of therapeutic molecules to αv integrins (16, 24, 26, 29), and that αv integrins are significantly over-expressed in bladder tumors in a stage and grade-dependent manner (35, 36), lend support to the hypothesis that this class of receptors may represent important molecular targets for toxin delivery to bladder cancer.

The approved clinical practice for the management of bladder cancer consists in transurethral resection of cancer lesions or by the removal of the entire organ (radical cystectomy), depending on the tumor grade and stage. Most of the times, a chemoprophylaxis regimen based on chemotherapeutics like platinum complexes or mitomycin C (MMC) is given either before surgery (neoadjuvant chemotherapy) or after surgery (adjuvant chemotherapy) to reduce the risk of cancer recurrence (33). In case of advanced or metastatic bladder tumor, immune checkpoint inhibitors (anti-PDL1 antibodies) and tyrosine kinase inhibitors (specific for FGFR) represent the most effective targeted options, showing promising results in the treatment of specific subtypes. However, the clinical outcome of these treatments strictly relies on the presence of an elevated immune signature or FGFR2/3 specific mutation (typical of patients with a luminal I bladder cancer subtype) (37, 38).

To recapitulate advanced bladder cancer features, we have tested the pharmacological efficacy of our recombinant protein, systemically administered, using syngeneic bladder cancer mouse models. At first we used a subcutaneous cancer model to determine the optimal dosage and found that RGD-SAP can inhibit the tumor growth in a dose-dependent manner. In this model, RGD-SAP was significantly more active than the CYS-SAP control, the latter being almost completely inactive. This suggest that RGD-SAP can actively target the tumor environment and exclude a “passive targeting” mechanism potentially related to the enhanced permeability of tumor tissues. Then, we used an orthotopically implanted tumor model (MB49) to assess the therapeutic effect of RGD-SAP alone and in combination with MMC. The MB49 orthotopic model of advanced bladder cancer is characterized by a logarithmic proliferation rate of the tumor mass, leading in several days to the formation of an inner necrotic area, causing a sudden drop of luminescence signal (27, 39). It is expected that the uptake of drugs in solid tumors is heterogeneous and the general distribution decreases with increasing tumor weight, since cells that are progressively distant to blood vessels and located in high-pressure regions constitute large areas of hypoxic, necrotic, or semi-necrotic tissue. Thus, this condition can limit an adequate penetration of drug administrated systemically, such as RGD-SAP, into the tumor mass. Interestingly, upon MMC pre-treatment, RGD-SAP reduced tumor growth compared to MMC alone, significantly increasing overall survival (80% of mice) and improving animal welfare. Notably, RGD-SAP has shown a low cytotoxic activity on MB49 cells *in vitro*, suggesting that its activity *in vivo* could be related to the targeting of microenvironment components as well. Indeed, αvβ3 is expressed by the endothelium in the neo-angiogenic blood vessels (16–19) and it represent a potential target of RGD-SAP.

Intra-tumoral heterogeneity represents a major obstacle to cancer therapeutics, including conventional chemotherapy, immunotherapy, and targeted therapies. Due to its potential effects on tumor cells and microenvironment, RGD-SAP may represent a good therapeutic tool for bladder cancer. In addition, by inhibiting proteins synthesis, SAP acts in a cell cycle independent manner, thus targeting both quiescent and rapidly dividing tumor cells. This feature makes it suitable to contrast both aggressively growing cancers and tumors with slower progression. RGD-SAP can be employed also in combination with other therapeutic options based on different mechanisms of action, e.g. inhibition of DNA synthesis, cell division, and signal transduction.

## Conclusions

Our study demonstrates that the fusion of RGD-4C to SAP enables specific delivery of the toxin to the tumor mass and enhances its anti-tumor activity in bladder cancer models without showing detectable side effects. The RGD-SAP may be potentially applicable to other solid tumors, especially in combination with other therapeutic agents to tackle tumor heterogeneity.

## Data Availability Statement

The raw data supporting the conclusions of this article will be made available by the authors, without undue reservation.

## Ethics Statement

The animal study was reviewed and approved by Institutional Animal Care and Use Committee, IACUC.

## Author Contributions

RV designed the project and supervised the work. SZ, CA, CM, OAB, and FC performed the experiments, acquired data, provide methods and reagents. RV, SZ, FC, MD, and AC discussed the results. SZ drafted the manuscript. RV, MD, FC, and AC revised the manuscript. All the authors approved the manuscript.

## Funding

This work was supported by the Italian Ministry of Health (GR-2011-02351220 to RV).

## Conflict of Interest

The authors declare that the research was conducted in the absence of any commercial or financial relationships that could be construed as a potential conflict of interest.

## Publisher’s Note

All claims expressed in this article are solely those of the authors and do not necessarily represent those of their affiliated organizations, or those of the publisher, the editors and the reviewers. Any product that may be evaluated in this article, or claim that may be made by its manufacturer, is not guaranteed or endorsed by the publisher.

## Supplementary Material

Supplementary Figure 1
**(A)** Quantitative analysis of growing tumor volume for each individual mouse treated with vehicle, RGD-SAP or CYS-SAP. **(B)** Quantitative analysis of growing tumor volume for each individual mouse treated with vehicle (0 mg/kg) or RGD-SAP 0.25, 0.5 and 0.75 mg/kg).
