# Supplementary figures and images for: A Novel RGD-4C-Saporin Conjugate Inhibits Tumor Growth in Mouse Models of Bladder Cancer
Source: Front Oncol. 2022 Apr 11;12:846958. doi: 10.3389/fonc.2022.846958 (PMC9035931; doi:10.3389/fonc.2022.846958)

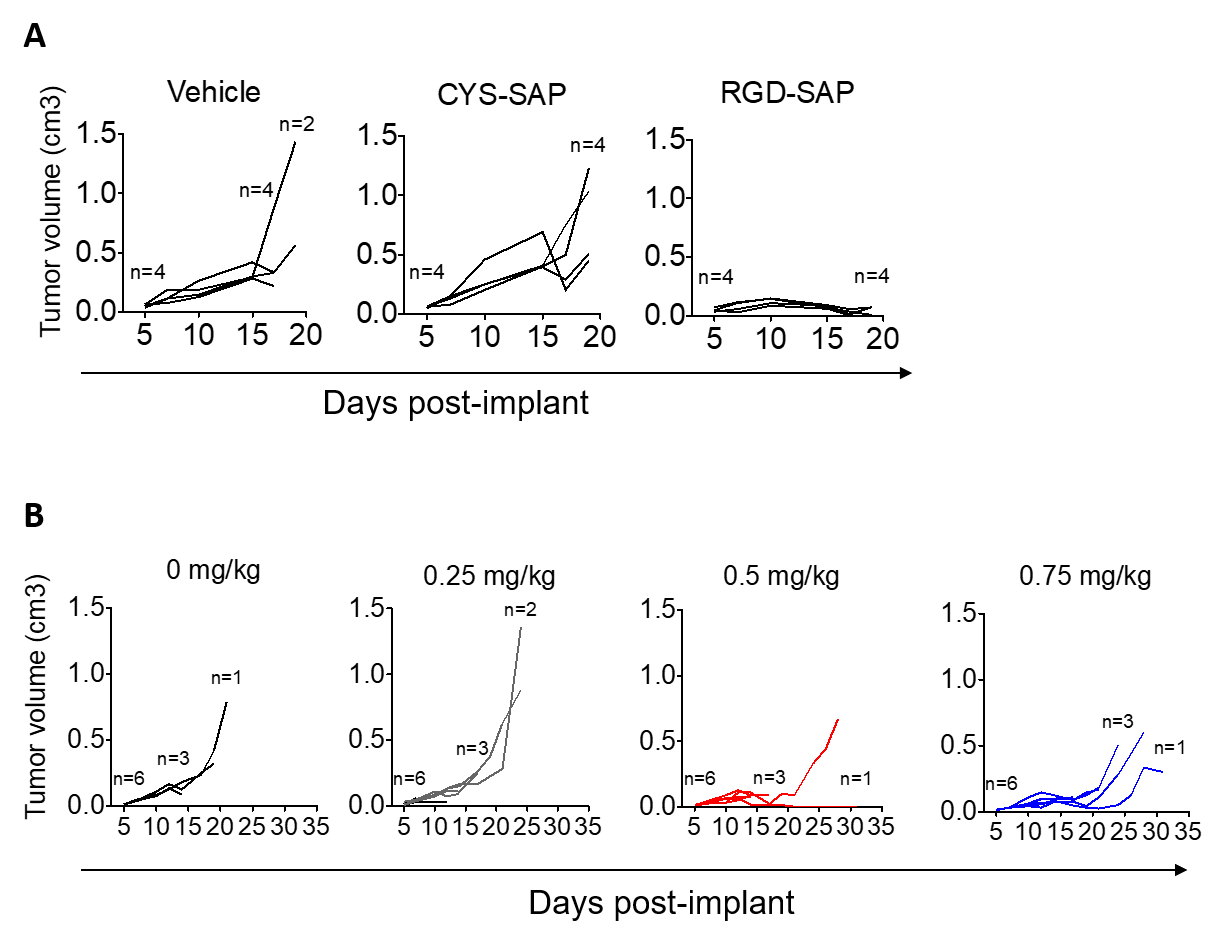

Supplement: Supplementary Figure 1 — (A) Quantitative analysis of growing tumor volume for each individual mouse treated with vehicle, RGD-SAP or CYS-SAP. (B) Quantitative analysis of growing tumor volume for each individual mouse treated with vehicle (0 mg/kg) or RGD-SAP 0.25, 0.5 and 0.75 mg/kg). [file Image_1.tif]
